# BioSolutions for Green Agriculture: Unveiling the Diverse Roles of Plant Growth-Promoting Rhizobacteria

**DOI:** 10.1155/2024/6181491

**Published:** 2024-08-29

**Authors:** Emmanuel Ehinmitan, Turoop Losenge, Edward Mamati, Victoria Ngumi, Patrick Juma, Beenzu Siamalube

**Affiliations:** ^1^ Department of Molecular Biology and Biotechnology Pan African University Institute for Basic Sciences, Technology and Innovation, P.O. Box 62000-00200, Nairobi, Kenya; ^2^ Department of Horticulture Jomo Kenyatta University of Agriculture and Technology, P.O. Box 62000-00200, Nairobi, Kenya; ^3^ Department of Botany Jomo Kenyatta University of Agriculture and Technology, P.O. Box 62000-00200, Nairobi, Kenya

## Abstract

The extensive use of chemical pesticides and fertilizers in conventional agriculture has raised significant environmental and health issues, including the emergence of resistant pests and pathogens. Plant growth-promoting rhizobacteria (PGPR) present a sustainable alternative, offering dual benefits as biofertilizers and biocontrol agents. This review delves into the mechanisms by which PGPR enhance plant growth, including nutrient solubilization, phytohormone production, and pathogen suppression. PGPR's commercial viability and application, particularly under abiotic stress conditions, are also examined. PGPR improves plant growth directly by enhancing nutrient uptake and producing growth-promoting substances and indirectly by inhibiting phytopathogens through mechanisms such as siderophore production and the secretion of lytic enzymes. Despite their potential, the commercialization of PGPR faces challenges, including strain specificity, formulation stability, and regulatory barriers. The review highlights the need for ongoing research to deepen our understanding of plant-microbe interactions and develop more robust PGPR formulations. Addressing these challenges will be crucial for integrating PGPR into mainstream agricultural practices and reducing reliance on synthetic agrochemicals. The successful adoption of PGPR could lead to more sustainable agricultural practices, promoting healthier crops and ecosystems.

## 1. Introduction

Chemical pesticides and fertilizers are essential for increasing yield and extending the shelf life of agricultural produce. Despite the undeniable positive impact of chemical pesticides in controlling plant diseases and pests, there is growing concern about their negative implications on human health. Chemical pesticides have adversely affected our immediate environment, including both marine and terrestrial habitats, leading to chronic toxicity in beneficial organisms. The persistence of chemical pesticides in the soil microenvironment affects a broad spectrum of microbes, including beneficial ones [1].

The intensive use of these chemicals over the years has led to the development of resistant pests and pathogens best exemplified by plant fungal disease resistance to azole, a broad-spectrum strobilurin [1]. This development of pesticide resistance has necessitated the use of higher dosages of chemical fungicides, thereby increasing the health- and environment-associated risks of these chemicals [2]. This situation has prompted the search for alternative chemical pesticides that are more potent with varying lethal modes of action while being milder to human and environmental health. The more stringent environmental impact assessment tests required before approving a new chemical pesticide for commercialization have made the development of new chemical pesticides daunting. In addition, the harmful effects on human health and the ecosystem have led to the withdrawal of about three-quarters of the legally available pesticides between 1993 and 2018, making getting a new chemical on the market more complicated than ever.

The negative implications of using chemical pesticides have led to a shift in agronomic practices, with a growing convergence towards biological control agents (BCAs) in disease management. The immense potential of BCAs as an alternative or complement to chemical control is making them increasingly popular as a significant component of integrated pest management (IPM) practices worldwide [3]. The use of biological control agents in the IPM system promotes the cultivation of healthy crops with minimal impact on human health and the agroecosystem.

Biological control utilizes naturally occurring organisms or their products in disease and pest management. Although the use of biological control is becoming increasingly popular, it is not without its challenges, primarily the lack of efficient and large-scale commercially available biocontrol agents in the open market [4]. Biocontrol agents are abundant in nature and include bacteria, protozoans, yeast, fungi, and viruses. The term rhizobacteria originates from the predominant isolation of BCAs from the soil rhizosphere, which contains many microbes due to its richness in nutrients [5]. These rhizobacteria are classified into three major groups: (a) beneficial rhizobacteria, (b) neutral rhizobacteria, and (c) deleterious rhizobacteria. The beneficial groups are known as plant growth-promoting rhizobacteria (PGPR) [5].

PGPR is of significant agricultural importance because of its role in sustainable agricultural practices. PGPR plays an important role in suppressing and managing phytodiseases, as well as improving essential nutrient availability in plants [6]. PGPR helps improve plant growth and health in a variety of ways. The direct mechanism involves either adding extra nutrients through microbes or making it easier for plants to absorb the nutrients that are already in the soil. PGPR can also act indirectly by eliminating or reducing the microbial load of phytopathogens in the rhizosphere [7]. They can also exhibit indirect biocontrol activity on pathogens through the secretion of various growth inhibitors, such as lytic enzymes, bacteriocins, siderophores, and antibiotics, or by inducing the host plant's inherent natural resistance [8, 9]. This review focuses on PGPR's mode of action, its promotion of plant growth under abiotic stress, and its commercialization limitations as biofertilizers and biocontrol agents.

### 1.1. Brief Historical Overview

The concept of using microorganisms to promote plant growth, specifically plant growth-promoting rhizobacteria (PGPR), has a rich history dating back to the late 19th century. Early researchers such as Martinus Beijerinck and Sergei Winogradsky explored soil microbes and their beneficial impacts on plants [10]. However, it was not until 1978 that the term “plant growth-promoting rhizobacteria” was formally introduced by Kloepper, who defined PGPR as bacteria that colonize plant roots and enhance plant growth through various mechanisms [11]. In the 1980s and 1990s, significant strides were made in understanding how PGPR promote plant growth. These mechanisms include nitrogen fixation, phosphate solubilization, production of phytohormones, and biocontrol of plant pathogens. During this period, numerous PGPR strains were isolated and characterized, including those from the genera *Azospirillum, Bacillus*, and *Pseudomonas* [12–14].

The commercial application of PGPR gained momentum in the late 1990s and early 2000s, with products such as Rhizo Plus and Serenade entering the market. These products were designed to enhance crop yields and protect plants from diseases [10, 15]. The 21st century has seen a surge in PGPR research, driven by the need for sustainable agricultural practices. Advances in molecular biology and genomics have provided a deeper understanding of the interactions between PGPR and plants at the genetic and molecular levels. This has led to the development of more effective PGPR strains and formulations. Recent studies have highlighted the role of PGPR in mitigating abiotic stresses such as drought, salinity, and heavy metals. For example, research has demonstrated that PGPR can enhance plant tolerance to drought by producing exopolysaccharides that improve soil structure and water retention [16, 17]. Overall, PGPR's historical development reflects a trajectory of increasing sophistication and application, aligning with broader trends in agricultural sustainability and biotechnological innovation.

## 2. Mode of Action of PGPR

**Figure 1:**
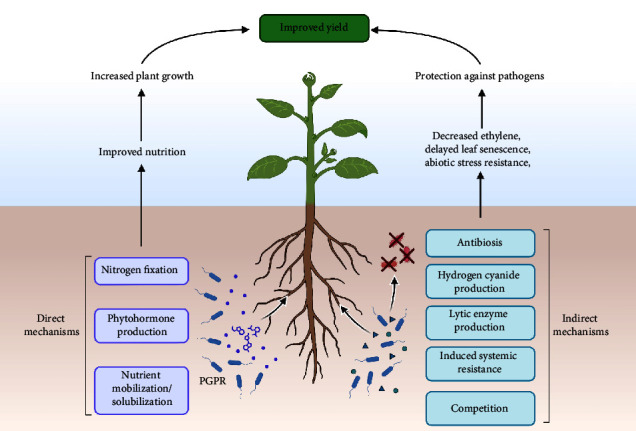
Schematic diagram depicting the mechanism of action of PGPR.

A detailed understanding of PGPR's mode of action is required to maximize its potential to improve plant productivity.  Figure 1 depicts PGPR's two mechanisms of action. PGPR has always operated through two modes of action: indirect and direct. As a general guideline, we refer to PGPR actions that occur outside the plant system as indirect. Conversely, we refer to activities within the plant that directly influence its metabolism as direct [18, 19].

The direct mode of action involves mechanisms that affect the balance of plant growth through growth regulators. The PGPR produce these regulators, which the plant system then integrates. Sometimes, the PGPR serves as a good source of phytohormones, as highlighted in Table 1, thus inducing plant growth and resistance to abiotic stress [38, 39].

The indirect mode of action leverages the plant's natural defense mechanism through the microbial signaling process. Furthermore, through indirect mechanisms, PGPR protects plants against environmental stress [40–42].

### 2.1. The Direct Mode of Action

The enhancement of plant growth in the absence of pathogens is categorized as direct PGPR action. This includes facilitating nutrient uptake and production of plant phytohormones, which impact both the rhizosphere and the phyllosphere.

#### 2.1.1. Solubilization of Inorganic Phosphate

Phosphorus is a major plant macronutrient, second only to nitrogen. Although phosphorus is abundant in nature, it is often insoluble and, therefore, unassimilable by the plant. Plants can only utilize phosphorus as mono- and dibasic phosphate. In the soil, PGPR breaks down complex insoluble organic phosphates such as aluminum phosphate, tricalcium phosphate, rock phosphate, and others into simpler soluble inorganic forms. This makes it easier for plants to get phosphate [18, 43].

Phosphatase-producing bacterial strains employ a variety of mechanisms to solubilize insoluble phosphate, mainly by releasing organic acids during the breakdown of carbohydrates. Examples of these organic acids include acetic, lactic, malic, gluconic, tartaric, butanoic, and succinic acids [43, 44]. These organic acids are effective chelators of Ca^2+^, releasing phosphate into its soluble phosphatic state. Various instrumental equipment, including gas-chromatography-mass spectrometry (GC-MS) and high-performance liquid chromatography (HPLC), has detected these acids [44]. Phosphate-solubilizing bacteria constitute about 20–40% of the total soil microbial community, with the majority located in the rhizosphere [44]. Researchers have reported that phosphate-utilizing PGPR exhibit greater metabolic activity than those isolated from other sources [33].

Plants often struggle to assimilate phosphate due to its reaction with cationic elements, such as iron, aluminum, and calcium-forming salts, during precipitation. In acidic soil, inorganic phosphates are salts of iron and aluminum, whereas calcium phosphate is predominant in calcareous soil [12]. Organic phosphate makes up most of the soluble phosphate in soils with a high organic matter content. Phytate comprises approximately 80% of this organic phosphate, although some microbes produce phytases that can hydrolyze this complex phosphate. However, this enzyme often forms complexes with metals such as aluminum, iron, and calcium [23, 45].

The best-known phosphate-solubilizing rhizobacteria are *Bacillus polymyxa, B. coagulans, and B. megaterium* [46]. Researchers have reported that *Pseudomonas fluorescence* is a powerful producer of gluconic acid, which aids in the solubilization of mineral phosphate [47]. Literature reports *Bacillus firmus, Rhizobium leguminosarum*, and *Ensifer meliloti* as excellent producers of organic acid 2-ketogluconic acid, which efficiently solubilizes mineral phosphate, while *Bacillus amyloliquefaciens* and *Bacillus licheniformis* are known to produce a mixture of isobutyric, isovaleric, and acetic acids [20, 22, 48].

#### 2.1.2. Nitrogen Fixation

Rhizobacteria strains capable of fixing atmospheric nitrogen are classified into two groups: symbiotic bacteria and free-living nitrogen fixers. Symbiotic bacteria are naturally associated with the roots of leguminous plants and penetrate the root to form nodules [49]. Examples include strains from the genera *Rhizobium, Bradyrhizobium, Sinorhizobium, Mesorhizobium,* and *Azorhizobium* [47].

The other group, free-living nitrogen fixers, forms close associations with the roots of crops, aiding in nitrogen fixation for the plants. This type of association is nonspecific and generally considered loose, unlike the symbiotic association [10]. Many PGPR strains found in *Azospirillum* and *Azotobacter* have become popular due to their ability to fix nitrogen both in the lab and in the environment. These strains have been used since the early 1900s and continue to be used today [50].

These organisms not only fix nitrogen but are also adept at producing phytohormones such as auxins, cytokinins, and gibberellic acid [51]. There have been reports of increased cereal crop productivity when *Azospirillum* and *Azotobacter* were used as agricultural inoculants. Currently, new strains of PGPR have been reported in the genus *Bacillus* [43, 52, 53]. Many *Methylobacterium* spp. have also been shown to participate in nitrogen fixation during interactions with plants. Specifically, *M. nodulans, M. symbioticum,* and *M. radiotoleran*s are reported as nitrogen-fixing bacteria [24, 54].

#### 2.1.3. Siderophore Production

Iron is an essential macronutrient for plants and acts as a coenzyme in crucial physiological processes such as nitrogen fixation, respiration, and photosynthesis. A deficiency in this major macronutrient will have a deleterious effect on the plant. Although iron is very abundant in the soil, it is not always easily accessible for plant and soil microorganisms [55, 56]. The oxidized form of iron, which is the predominant form, readily forms insoluble hydroxides and oxides. These insoluble oxides are inassimilable by plants and microbes. However, plants have strategized two equally efficient methods for iron absorption.

In the first method, the plant secretes an organic acid that chelates the iron, making it soluble. This acid then diffuses toward the plant root, where it is reduced and absorbed. The second is more direct: Some plant species can absorb iron in its complex oxide and hydroxide forms and reduce it within the plant system [57, 58]. Many PGPRs produce siderophore, a great chelating agent present in the soil rhizosphere. These siderophores trigger the movement of soil iron towards improved iron uptake by the plant [37, 59].

Siderophores can bind reversibly with iron because of the chemical properties of their functional group and their low molecular weight (less than 1 kDa). The most common groups of siderophores are catechols, carboxylates, and hydroxamates. The structures offer an ideal distance between molecules for efficient binding to iron [60]. The siderophore concentration in the soil is often relatively low (10–30 mM). PGPR strains of *Pseudomonas* are excellent producers of siderophores such as *Pseudomonas aeruginosa and Pseudomonas fluorescence* [61]. These PGPR strains release their siderophores (pyoverdine and pyochelin) to gain competitive advantages over other rhizosphere-dwelling microbes because they possess potent antibiotic properties [7].

Several studies have demonstrated the role of siderophores in plant growth promotion [62, 63]. Siderophore-producing PGPR strains help improve plant growth through enhanced iron uptake while deterring pathogenic fungi growth through antibiosis. Furthermore, siderophore can cause iron deficiency in fungal phytopathogens by limiting iron supply because these pathogens are incapable of utilizing the iron-siderophore complex [64].

#### 2.1.4. Production of Phytohormones

Phytohormones are also known as plant hormones, whose examples include auxin, cytokinin, gibberellins, ethylene, abscisic acid, brassinosteroids, and jasmonates. Microorganisms in the bulk soil can produce these hormones, but rhizobacteria do so more efficiently [43]. The plant can easily assimilate microbially produced phytohormones. These hormones regulate and modulate processes in the plant system, such as cell division, enlargement, and extension of leguminous and nonleguminous roots [65, 66].

*(1) Cytokinin*. PGPR produces cytokinin, a primary class of phytohormone. The literature has extensively discussed the roles of cytokinin, abscisic acid, gibberellin, and auxins in enhancing plant growth and yield [67–69]. When applied exogenously, cytokinin has a similar effect on plants as auxin. It improves root growth, cell division, and root hair formation. It helps initiate shoot formation and inhibit root elongation, among other physiological responses [68]. Cytokinin is an aminopurine with a chemical substitution at the N6 position. When this phytohormone is applied to plants, it improves developmental and physiological processes. Examples of development processes include leaf branching, leaf extension, root growth, embryo vasculature development, promotion of seed germination, nutritional signaling, and delayed senescence [70]. Reports indicate that cytokinin significantly influences chlorophyll production [71].

Researchers have extensively studied the microbial production of cytokinin by PGPR, resulting in the isolation, identification, and characterization of several strains. These strains have been isolated from a vast number of genera, such as *Bacillus, Azospirillum*, and *Pseudomonas*, from various plants such as wheat, canola, beans, and barley [12, 72, 73]. A PGPR strain, *B. megaterium*, has been reported to improve tea growth through enhanced production of cytokinin [74]. Other PGPR strains from the genera *Xanthomonas, Escherichia, Klebsiella, and Proteus* can biosynthesize cytokinin, as reported in the literature [75–77].

Biochemically, cytokinin exists primarily as either kinetin or zeatin. In both cases, an isoprenoid replaces the N6 of the adenine backbone. In PGPR, zeatin is synthesized using the adenosine monophosphate, or tRNA, pathways. *Bradyrhizobium japonicum and Bradyrhizobium leguminosarum* have been reported to produce a cytokinin-like compound in their culture filtrate [78]. This cytokinin-like substance was later identified as zeatin. Two PGPR strains of *Rhizobium* spp *IC3342 and ANU240* were reported to produce large amounts of zeatin and isopentenyl adenine [79]. Cytokinin produced by *Pseudomonas G20-18* was also said to have exhibited growth-promoting effects on radish and wheat plants. Over 30 cytokinin-like substances have been identified from microbes associated with plants, and these microorganisms can produce the phytohormone at varying concentrations [80].

*(2) Auxin*. PGPR commonly produces auxin as indole-3-acetic acid (IAA), a phytohormone integral to many plant's metabolic processes, such as apical dominance, cell division, cell elongation, and tissue differentiation. Researchers have thoroughly investigated the biosynthesis and mechanism of action of this crucial phytohormone. Scientific research indicates that coinoculating plants with auxin-producing PGPR will improve plant metabolic performance, growth, and yield [81].

A similar study revealed that auxin induces rapid (short-term) and nonrapid (long-term) responses in plants. Rapid responses typically include cell elongation, while nonrapid responses encompass cell differentiation and cell division. According to the research conducted by the authors in [80], plants grown under long-term auxin treatment develop a better root system, enhancing their nutrient uptake capabilities and overall growth.

Over 80% of PGPR can produce auxin. When the hormone combines with the indigenous plant auxin, it triggers a combinatory effect that promotes plant growth. While auxin enhances all plant parts, it notably improves the root system [82]. These roots become larger and heavier and exhibit more branching, increasing their soil contact surface area. The improved root features enhance the root system's efficiency in nutrient search, thereby improving the plant's growth capacity and nutrient pool [83]. Auxin has proven efficient in the redifferentiation of roots from stem tissue [84]. Auxin produced by bacteria strains of the rhizoplane and endophytic niches has been reported to improve plant growth significantly [81].

Different PGPR strains synthesize IAA using various pathways. These pathways can be divided into two major categories: (1) the L-tryptophan-dependent pathway and (2) the L-tryptophan-independent pathway. Root exudates contain L-tryptophan, which PGPR utilizes in the L-dependent pathway. While some PGPR strains possess L-tryptophan-independent pathways, they are less common. For instance, *Azospirillum brasiliense* produces 90% of its auxin through the L-tryptophan-independent pathway and only 10% through the L-tryptophan-dependent pathway. The detailed biochemistry of these biosynthetic pathways remains largely unknown [10, 55, 83]. The three L-tryptophan-dependent pathways are (1) the indole-3-pyruvic acid (IPyA) pathway, (2) the indole-3-acetamide (IAM) pathway, and (3) the tryptamine pathway [85].

*(3) Gibberellin*. Gibberellins, a large group of phytohormones, consist of 130 structurally diverse molecules. This hormone affects many plant development processes, such as stem elongation, flowering, fruit set, and seed germination [86, 87]. Only four gibberellins (GA 20, GA 4, GA 3, and GA 1) have been identified from seven bacteria strains. In addition, researchers have identified and characterized 28 gibberellins from 7 fungi species and 128 plant species [88]. Strains from the genus *Bacillus* are generally nonproducers of gibberellins, except for some known strains, namely, *Bacillus licheniformis* and *Bacillus pumilus* [83].

This group of phytohormones can translocate from the root to the plant's aerial region, positively affecting plant growth. Microbes can produce gibberellins, which consist of a 19–20 carbon atom backbone [83, 86, 88]. GAs are more efficient for aerial plant growth, especially if the PGPR also produces auxin. This enhances the root system, thereby facilitating growth in the aerial part and improving the nutrient supply.

Researchers first documented GAs produced by PGPR through characterization using some physiochemical methods [72, 89]. They carefully examined *Ensifer meliloti* grown on gnotobiotic culture and found 4 GAs: GA 20, GA 9, GA 4, and GA 1. Many strains of bacteria have been reported to produce GAs, although the methods used for many characterizations, such as *in vitro* bioassay and thin-layer chromatography, are unreliable [83, 90]. Gas-chromatography-mass spectrometry (GCMS), a more reliable physiochemical method, confirmed the production of GAs in *Herbaspirillum seropedicae* and some *Bacillus* spp. [91].

The biosynthesis pathways of gibberellins in fungi are similar to those of higher plants; however, the required enzymes differ. Ongoing exploration into the mode of action of gibberellins produced by PGPR emphasizes their ability to enhance seed germination. While PGPR capable of producing gibberellins are termed beneficial microbes, fungi that can produce the substance are considered phytopathogens [70].

**Figure 2:**
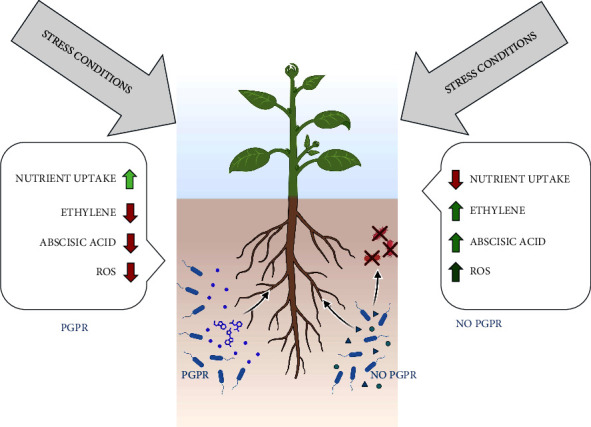
The mechanism of improvement of crop tolerance to abiotic stress. The green arrow signifies the activation effect whereas the red arrow indicates effects' reduction.

*(4) Other Phytohormones*. In addition to auxin, gibberellin, and cytokinin, PGPR can produce several other phytohormones that play significant roles in plant growth, development, and stress responses. Some strains of PGPR have been reported to produce abscisic acid (ABA), a phytohormone involved in various physiological processes such as seed dormancy, stomatal regulation, and responses to abiotic stresses [92]. ABA production by PGPR can contribute to enhanced stress tolerance in host plants by regulating water balance as depicted in Figure 2 [93]. Certain PGPR strains can also produce ethylene, a gaseous phytohormone that regulates diverse aspects of plant growth and development, including fruit ripening, leaf abscission, and responses to biotic and abiotic stress [94]. PGPR-produced ethylene modulates plant responses to acute environmental change and promotes adaptation to changing conditions [95].

PGPR jasmonic acid has also been identified as a key mediator of plant defense responses against pathogens, as well as a regulator of growth and development processes [96]. Some other PGPR strains have been shown to synthesize salicylic acid (SA), a phytohormone known for its role in systemic-acquired resistance against pathogens and its involvement in various signaling pathways related to plant defense [97]. Furthermore, Some PGPR can also synthesize polyamines such as spermidine and spermine, which are involved in various physiological processes including cell division, differentiation, and stress response [98]. These additional phytohormones further highlight the diverse array of signaling molecules produced by PGPR, underscoring their importance in mediating plant-microbe interactions and promoting plant growth.

### 2.2. Indirect Mechanism of Action by PGPR

**Figure 3:**
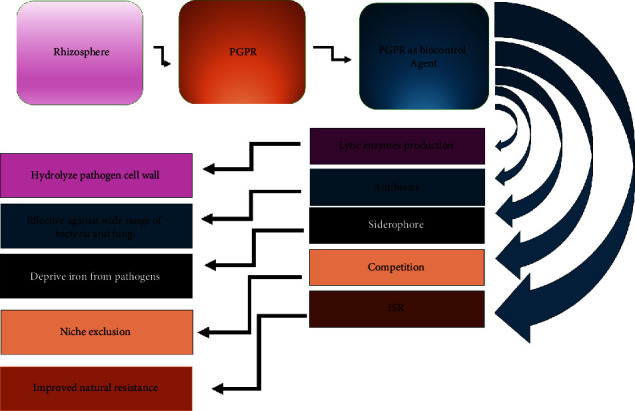
PGPR mode of action as a biocontrol agent.

The major indirect mechanism by which rhizobacteria promote plant growth is through their role as biocontrol agents (Figure 3). Some of the biocontrol activities exhibited by PGPR include competition for nutrients, niche exclusion, induced systemic resistance, and the production of antifungal and antibacterial metabolites, as highlighted in Table 2.

#### 2.2.1. Competition

For a PGPR strain to be an efficient biocontrol agent (BCA), it must persevere in the soil and colonize the plant root even under the harsh abiotic conditions of the field. Therefore, the BCA must successfully colonize the plant root and compete with other root-dwelling microbes for nutrients and niches. Competition is a critical trait of a good BCA. It is well-documented that PGPR, with their rapid colonization of the root, efficiently protect the plant from pathogenic microbes. They do this by preventing pathogens from attaching and limiting nutrient availability to the pathogen [124, 125]. In addition, the BCA also produces secondary metabolites that are harmful to the pathogens [126].

Interestingly, some root exudates contain secondary metabolites with deleterious antimicrobial properties to most microorganisms. However, with the necessary detoxification machinery, PGPR can gain a competitive advantage and occupy the ecological niche. In summary, PGPR competence depends on their ability to adapt to and maximize potentially harsh microenvironmental conditions [127].

#### 2.2.2. Production of Hydrogen Cyanide (HCN)

PGPR is known to produce HCN through de novo synthesis from smaller precursor compounds. This HCN possesses antimicrobial properties, making it useful for suppressing diseases [128, 129]. HCN is harmful to a critical enzyme (cytochrome oxidase) of metabolic respiration, thereby distorting the electron transport chain, which ultimately results in cell's death [130, 131]. The unique HCN-producing ability of certain PGPR strains makes them potent biocontrol agents against phytopathogens. Notably, many strains of *Pseudomonas* and *Bacillus* are exceptional HCN producers [131]. In addition, bacterial cyanogenesis has been reported in PGPR strains of various genera, including *Burkholderia*, *Rhizobia,* and *Chromobacterium*, highlighting the diversity and potential of these biocontrol agents [132].

Similarly, it has been observed that *Pseudomonas*, particularly the fluorescent strain, are efficient HCN producers as part of their secondary metabolism [133]. This is evidenced by the 45% inhibition of Fusarium wilt in a tomato plant inoculated with *Brevundimonas oleic Prd2* [134]. Furthermore, *Pseudomonas fluorescence LRB3W1* and Pseudomonas sp. *RhB-12* have been reported to be highly effective against *Fusarium oxysporum f. sp. lycopersici* [135].

#### 2.2.3. Production of Hydrolytic Enzymes by PGPR

PGPR can exhibit biocontrol activity by producing enzymes that degrade the pathogen's cell wall. Enzymes such as glucanase, protease, chitinase, and cellulase produced by PGPR strains serve as biocontrol agents by destroying the cell wall of fungal phytopathogen [31, 136–138].

Chitinase is a lytic enzyme that degrades the building blocks of chitin (*β* 1-4-N-acetyl-glucosamine). Chitin is an essential component of the cell wall of fungi. It has been reported that the fungal cell wall in *Fusarium oxysporum* can be degraded by *β* 1-4 glucanase produced by PGPR strains of *Streptomyces and Paenibacillus* [139, 140]. Similarly, a wide array of bacteria, including *Serratia marcescens, Pseudomonas fluorescence, Pseudomonas aeruginosa, Enterobacter agglomerans, Bacillus thuringiensis, Bacillus subtilis, Bacillus cereus,* and *Bacillus circulans*, have been found to possess the potent chitinolytic activity, underscoring the widespread nature of this biocontrol mechanism [140]. This group of lytic enzymes shows their deleterious capabilities by destroying the structural integrity of their target pathogen.

Studies have shown that *Serratia marcescens* can produce chitinase with potent biocontrol, antifungal, and chitinolytic activities against known fungal phytopathogens, such as *Fusarium oxysporum and Rhizoctonia solani* [141]. Following inoculation with PGPR, the mycelia of these fungal pathogens exhibited striking deformities, such as hyphae bursting, curling, and swelling when viewed under a microscope, providing tangible evidence of the biocontrol process in action [142].

#### 2.2.4. Antibiotic Production by PGPR

The use of natural enemies of phytopathogens for crops of economic importance has been a promising alternative to chemical pesticides. Numerous PGPR strains of *Bacillus* and *Pseudomonas* have been identified for their ability to suppress pathogens through antibiotic secretion. These PGPR strains produce secondary extracellular molecules that can effectively combat plant pathogens even at low concentrations. For instance, the genus *Bacillus* produces an extracellular compound with well-defined antibacterial and antifungal properties. The potential of these antibiotic compounds, such as sublancin, subtilosin A, TasA, and subtilin, and others from families of iturin, fengycin, and surfactin, is immense [143, 144].

Strains of *Pseudomonas aeruginosa and P. fluorescence* are known to produce extracellular compounds with potent antibacterial properties [145]. These compounds are not just efficient biocontrol agents against bacteria but they also have a wide range of applications. They are antiviral, antitumor, antihelminthic, antioxidant, phytotoxic, and cytotoxic. Their versatility and potential for various uses make them a fascinating area of study [144]. Examples of these extracellular metabolites are numerous and diverse, including cepaciamide, phenazine-1-carboxamide (PCN), 2-4-diacetyl phloroglucinol (DAPG), pyoluteorin, oomycinA, pyrrolnitrin, butyrolactone, ecomycins, and cepafungins [146].

#### 2.2.5. Induced Systemic Resistance (ISR)

PGPR often adopt an indirect mechanism to protect plants through induced systemic resistance. It can be defined as a process where microbial inoculation treatment elicits the host plant's inherent defense mechanism, despite often being spatially separated from the inducing agent [124]. The nonspecificity of ISR is a key advantage, as it provides resistance to a wide range of pathogens that a plant may encounter under natural conditions. This versatility makes ISR a valuable tool in plant disease resistance [28].

It has been reported that some PGPR strains of *Pseudomonas* trigger ISR in crops such as wheat and radish. The elicitor is the O-antigenic side chain of the bacterial lipopolysaccharide's outer membrane. In addition, the pseudobactin siderophore of some PGPR strains has been reported to trigger ISR in wheat and tobacco plants. Similarly, pseudomanine siderophore produced in some other strains of *Pseudomonas* has been demonstrated to induce the production of salicylic acid in some plants (radish and wheat), which elicits ISR, thereby improving the plant defense mechanism [147, 148]. By inoculating the plant's root with ISR eliciting rhizobacteria, we can trigger a comprehensive effect that extends beyond the root to all parts of the plant system. This significantly enhances the plant's resistance to disease development, providing a robust defense mechanism [27].

### 2.3. PGPR Modulates Plant Stress Makers under Abiotic Stress Conditions

**Figure 4:**
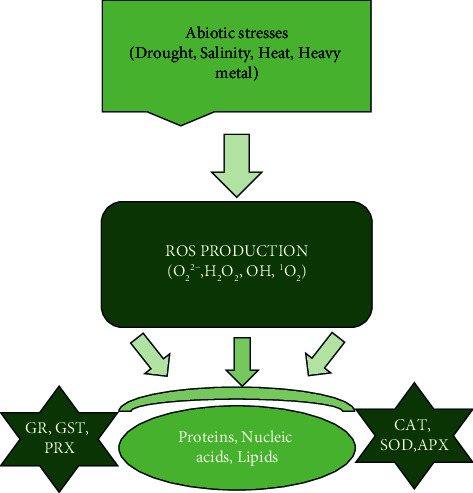
PGPR antioxidant enzymes' roles in ROS scavenging.

Abiotic stress, including salinity, flooding, extreme temperature, heavy metal contamination, and drought, contributes to about 30% of the total yield loss of crops worldwide. Among these, salinity is particularly harmful, disrupting many essential processes of plant metabolism such as respiration, photosynthesis, and protein synthesis. This disruption significantly affects crop yield, highlighting the challenges that plants face in their growth and development [149]. Salinity, a major contributor to abiotic stress, induces nutrient deficiencies in plants primarily due to the excessive absorption of Na^+^, which hampers the uptake of other crucial ions [150]. Abiotic stress exerts hyperosmotic or ionic stress, which ultimately results in biochemical oxidative stress. Oxidative stress stems from the production of reactive oxygen species (ROS) such as superoxide anion, hydrogen peroxide, hydroxyl radical, singlet oxygen, peroxyl radical, alkoxyl radical, and hypochlorous acid in the plant biochemical pathways [151, 152]. These ROS damage the plant lipids and destroy other plant biomolecules, such as nucleic acids and proteins [151]. To counteract these highly reactive oxygen species, plants have developed an impressive antioxidant system. This system, illustrated in Figure 4, is a key factor in enhancing crop tolerance to abiotic stress. The production of ROS-degrading enzymes such as glutathione reductase (GR), catalase (CAT), ascorbate peroxidase (APX), glutathione-S-transferase (GST), peroxiredoxins (PRX) and superoxide dismutase (SOD) in various organelles (mitochondria and chloroplast) demonstrates the plant's ability to adapt and survive under challenging conditions [152].

It is well-documented that plants under salinity stress experience a surge in ROS levels. However, the plant's ROS-scavenging mechanisms also show a corresponding increase, indicating the plant's ability to counteract the stress [153]. PGPR inoculation of plants under abiotic stress is a significant factor in modulating the production of ROS-scavenging enzymes. For instance, the inoculation of the PGPR strain (*Bacillus cereus*) with tomato under physiochemical stress led to the production of CAT, SOD, and APX, enhancing the host's defense mechanism. The more these scavenging enzymes are produced, the better the defense [100]. Another key aspect is the role of L-proline, an amino acid that accumulates excessively in plants under abiotic stress, such as salinity. L-proline plays a crucial role in protecting the folded structure of proteins from denaturation and interacts with membrane phospholipids, stabilizing the plant cell walls. In addition, L-proline serves as both an energy and nitrogen reservoir and plays a vital role in maintaining osmotic equilibrium in plants. The foliar application of a PGPR strain (*Klebsiella spp.*) has been shown to increase yield in wetland rice under severe drought stress, highlighting the benefits of PGPR in sustainable agriculture [114]. Not only was the stress ameliorated, but nutrient uptake and L-proline bioaccumulation also improved. Therefore, PGPR strains hold the potential to significantly alleviate abiotic stress by eliciting ROS-scavenging enzymes or overproducing L-proline.

The performance of PGPR is significantly influenced by temperature and climatic conditions. For instance, suboptimal temperatures can directly affect the metabolic activity and growth rate of PGPR. Optimal temperatures promote their proliferation and activity, enhancing plant nutrient uptake, nitrogen fixation, or phytohormone production. Conversely, extreme temperatures can inhibit PGPR activity or even cause cell death, reducing their beneficial effects on plants [154]. Environmental factors such as humidity, rainfall, and seasonal changes play a crucial role in the performance of PGPR. Adequate moisture levels are essential for PGPR survival and mobility in the soil. Dry conditions can lead to desiccation and reduced efficacy, while excessive moisture may cause oxygen depletion, affecting their aerobic metabolic processes. Seasonal variations can alter PGPR communities, as different strains have varied tolerances to climatic fluctuations. Therefore, understanding and optimizing these environmental factors are crucial for maximizing the benefits of PGPR in sustainable agriculture [155].

### 2.4. Production of 1-Aminocyclopropane-1-Carboxylic Deaminase

Ethylene, a crucial phytohormone, plays a significant role in the plant ripening process. However, this hormone is overproduced during abiotic stress conditions such as salinity, flooding, and drought, leading to harmful effects [81]. Excessive accumulation of ethylene within a plant system can lead to detrimental effects such as leaf senescence, flower wilting, leaf abscission, and chlorosis. These effects underscore the importance of understanding the biochemical synthesis of ethylene, with 1-aminocyclopropane-1-carboxylate (ACC) as a precursor [10]. Many PGPRs have the potential to produce ACC deaminase, an enzyme that degrades ACC, thereby preventing ethylene formation. When plants are inoculated with ACC deaminase-producing PGPR, ACC is degraded into *α*-ketobutyrate and ammonia, offering a promising solution for protecting plants under severe abiotic stress [38].

It has been reported that *Achromobacter piechaudii* ARV8 helps increase tomato plant's stress tolerance to drought and salinity [100]. Likewise, it has been demonstrated that ACC deaminase-producing PGPR increases plant tolerance to abiotic stress such as heavy metal toxicity, flooding, drought, temperature, and UV exposure [156–158].

## 3. Commercialization of PGPR as Biofertilizers and Biocontrol Agents

Although PGPR as a bioinoculant has shown promise, there are still daunting challenges to overcome before full exploitation. The application of PGPR as a biostimulant in plants is centuries old, especially as a natural inhabitant of legumes and cereal root microbiomes. Many new strains of PGPR have been isolated, identified, and characterized using specific laboratory assays that rely on the mode of action of the PGPR. These assays, such as nitrogen fixation, calcium phosphate solubilization, and auxin synthesis, are significant as they provide a deeper understanding of how PGPR functions [43, 159].

The commercialization of PGPR as a biological product is a multifaceted process. These processes are (a) isolation, identification, and screening of potential plant growth-promoting properties in their natural environment; (b) field trial for efficiency/efficacy of the bioproduct; (c) mass production of the product; (d) delivery; (e) environment impact assessment test; (f) registration of the product with appropriate authorities; and (g) commercial release of the bioproduct to the market.

To successfully develop and commercialize BCA, knowledge of the phytopathogen species, pathogen's resistance, epidemiology of the disease, the prevailing abiotic condition under which the BCA will be used, and the type of host the pathogen attacks is required [128]. It has been reported that commercial bioproducts of *Pseudomonas aeruginosa 2apa and Bacillus subtilis PSIRB2* significantly reduced the incidence of early blight and Fusarium wilt in tomatoes [160, 161]. Likewise, the talc-formulated product *of Pseudomonas fluorescence* delivered through vermicompost and organic manure has demonstrated repression of *Fusarium oxysporum* in a field experiment, showing promising results in disease suppression [162].

Many methods have been used to formulate fungal and bacterial BCA and biofertilizers. Fungal biofertilizers/BCA often come in granular or powdery form. Also, gel formulations are used to develop both bacterial and fungal-based BCA. PGPR-based BCA comes in different forms; see Table 3 for a detailed outline of some commercially available PGPR strains. The sporulating properties of gram-positive PGPR are often exploited in formulating their biological products. Spores are resistant to heat and desiccation, making them ideal for formulating dry and stable talc/powder products. Alternatively, some PGPRs are marketed in suspension oil, which helps preserve the product by preventing microbial respiration and extending the bioproduct's shelf life [177].

For all the potential of BCAs in sustainable agriculture, their full utilization in agricultural practices is still limited because of the lack of in-depth knowledge of plant-microbe interactions, especially under the field's unpredictable and uncontrolled environmental conditions. Notwithstanding, some progress has been made in understanding plant-microbe interactions better. For a PGPR inoculum to be deemed ideal for field use, it must possess the following attributes: (a) it must be able to colonize the root of the plant efficiently, (b) it must be able to persist on the root even under unfavorable environmental conditions, and (c) the inoculum must not be hazardous to human health and the environment. Many promising PGPR strains perform poorly in field trials because they are incapable of colonizing the desired plant root microenvironment [178].

### 3.1. Limitations in the PGPR-Based Product Commercialization Success

According to data reported [179], crops are affected by over 67,000 pests and diseases, causing about a 40% yield loss worldwide. Biocontrol has been increasingly popular as a substitute for harmful chemicals that threaten human health and the environment. However, compared to chemical pesticides, very few biocontrol products are commercially registered and readily available [180].

The carrier material in PGPR-based products plays a pivotal role in their efficiency and survival in the field. This active component is crucial for maintaining the product's stability, application efficacy, and safety in field usage. PGPR-based bioproducts come in various forms, each defined by the properties of the carrier material, such as granules, dust, wettable powder, or oil-based form. However, these product forms can be broadly classified into two categories: dry powder and liquid suspension. The carriers used in these formulations prevent desiccation, which can be fatal to the inoculum, and create an ideal microenvironment for bacterial growth in the field.

The commercial success of PGPR as an efficient biofertilizer and biocontrol agent depends on many factors, such as the broad-spectrum action of the product, available market, shelf life, health and environmental safety issues, and synergy between industries and research organizations. The factors limiting the commercialization success of PGPR bioproducts are discussed as follows.

#### 3.1.1. Specificity Issue of the PGPR Bioproduct

The limited specificity and efficacy of commercially available PGPR products have significantly hindered their acceptance and usage. The ability of these products to colonize the root microenvironment depends on their specificity. Although some PGPR strains display broad specificity, most commercially available PGPR products have a limited range. For instance, efficient PGPR strains used in maize cultivation might exhibit lower or no efficacy in wetland rice cultivation. This phenomenon can result from the varying growth conditions of the two crops, compelling developers to produce different consortia of strains for various crops, which is not economical [181].

#### 3.1.2. Gram-Positive and Gram-Negative Dichotomy in PGPR Formulation

Many commercially available strains on the market are gram-positive bacteria due to their high population propensity and greater stability compared to gram-negative bacteria [182]. However, several gram-negative strains are also marketed commercially despite the challenges in formulating them. Despite the disadvantages of gram-negative strains, they have a significant advantage over gram-positive strains: they can persist longer in the soil without the need for reinoculation. To address the short life span of formulations containing gram-negative strains, the microbial load of the product should be increased. Even as the viable number of microbes declines by the time the product reaches farmers, enough will remain viable for effective seeding.

#### 3.1.3. Farmers' Acceptability

Chemical pesticides and fertilizers have been commercially available for almost a century with their effectiveness and efficacy well known to farmers. The effects of these chemicals on crops are immediate in terms of disease/pest repression and nutrient availability. Another advantage of chemicals over biological products from the farmers' viewpoint is the small amount of chemical use and the resultant higher benefits. The practical evidence of the efficacy and effectiveness of PGPR products is not widespread, thereby creating doubts in the minds of some farmers [183]. Another challenge to PGPR acceptability is the high cost due to its relative newness. Furthermore, growers of fruits and vegetables who place much value on the cosmetic appearance of their products are unlikely to adopt a newer technology because of the fear of cosmetic damage to the produce [184]. However, the negative trends of farmer acceptability are changing, with surveys among farmers using PGPR products revealing high satisfaction and willingness to continue using these bioinoculants. In fact, 72.73% of farmers expressed interest in further integrating PGPR into their farming practices [185].

#### 3.1.4. Survival and Stability Issues of the PGPR Formulation

The bioformulation of gram-negative PGPR strains has proven problematic due to their inability to produce spores [186]. The formulation process plays an essential role in the stability of PGPR strains, providing protection against desiccation at the target zone and improving delivery and efficacy against the target. However, the details of the formulation process remain a trade secret, resulting in limited scientific advancement in improving the process. The compatibility of microbes in a consortia formulation is also problematic. Many effective individual PGPR strains are incompatible in a consortium, creating a selection challenge for developers. Even if synergism is achieved among the strains in a consortium, other daunting factors must still be considered. These include the toxicity of the formulation to the host plant, human and animal health, the ecosystem, compatibility with other agrochemicals, efficacy, consistency in controlling plant disease, and above all, the cost-effectiveness of the product.

#### 3.1.5. Reinoculation and Handling of PGPR

PGPR strain bioformulations are living organisms, so care must be taken in their selection, preparation, and packaging. The formulation is embedded in a carrier material that facilitates the inoculum's ability to self-reproduce and helps colonize the target upon delivery. Another challenge affecting the commercialization of PGPR products is the mishandling by farmers, which can affect the product's efficacy. In addition, a significant obstacle to the successful commercialization of PGPR is the need for frequent reinoculation, as the strains cannot persist indefinitely in the soil.

#### 3.1.6. Unpredictability of the PGPR Strains in the Field Abiotic Environment

For PGPR to fulfill its potential in nutrient availability and pathogen repression, it must first colonize, establish, and proliferate within the ecological microenvironment [187]. Therefore, the competitive process depends on the characteristics of both the PGPR strains and the host. A specific population density is required to achieve efficacy for PGPR bioproducts. In addition, PGPR strains are often specific to particular cultivars or hybrids of the same crop species. The primary issue with PGPR bioproducts is the unpredictability of the strains under various abiotic environmental conditions, such as humidity, soil type, soil pH, rainfall, and temperature. These uncontrollable and often unpredictable environmental factors can cause the effectiveness of PGPR strains to vary from field to field.

#### 3.1.7. Regulatory Challenges: A Comparative Analysis

The commercialization of plant growth-promoting rhizobacteria (PGPR) faces significant regulatory challenges across different countries, which impact their development and market penetration. This subsection provides a comparative analysis of regulatory frameworks from representative countries in various continents to illustrate the global regulatory landscape.

*(1) North America: United States and Canada*. In the United States, the Environmental Protection Agency (EPA) oversees the registration and regulation of biopesticides, including PGPR. The registration process involves rigorous testing for efficacy, environmental impact, and human health safety, making it a lengthy and costly process [188]. Similarly, Canada, through the Pest Management Regulatory Agency (PMRA), requires extensive data on the environmental and health impacts of biopesticides before approval. These stringent requirements not only ensure safety but also pose a significant barrier for new products entering the market [189].

*(2) Europe: European Union*. The European Union (EU) has one of the most stringent regulatory frameworks for biopesticides. The European Food Safety Authority (EFSA) conducts comprehensive risk assessments, focusing on environmental and human health impacts. The approval process involves multiple stages, including scientific evaluation and public consultation, which can take several years to complete. Despite these challenges, the EU is committed to promoting sustainable agriculture through the use of biopesticides, reflected in its support for research and innovation in this field [190].

*(3) Asia: China and India*. China and India represent significant markets for PGPR but have different regulatory approaches. In China, the Ministry of Agriculture and Rural Affairs oversees the registration of biopesticides, with an emphasis on efficacy and environmental safety. However, regulatory processes can be inconsistent, posing challenges for international companies. India, regulated by the Central Insecticides Board and Registration Committee (CIBRC), has a more streamlined process but faces challenges related to enforcement and compliance, impacting the overall effectiveness of regulations [191, 192].

*(4) Africa: Kenya and South Africa*. In Africa, regulatory frameworks are less developed compared to other regions. Kenya and South Africa have made strides in regulating biopesticides through their respective agricultural ministries. However, the lack of standardized regulations and limited resources for enforcement pose significant challenges. Efforts are underway to harmonize regulations across the continent to facilitate easier market access and ensure product safety [193, 194].

*(5) South America: Brazil and Argentina*. Brazil and Argentina are leading agricultural producers in South America, with regulatory frameworks that support the use of biopesticides. Brazil's Ministry of Agriculture, Livestock, and Supply (MAPA) and Argentina's National Food Safety and Quality Service (SENASA) have established clear guidelines for biopesticide registration, focusing on safety and efficacy. These countries are actively promoting biopesticides as part of their sustainable agriculture initiatives [195, 196].

*(6) Australia*. Australia has a well-established regulatory framework for biopesticides overseen by the Australian Pesticides and Veterinary Medicines Authority (APVMA). The APVMA requires extensive data on the safety, efficacy, and environmental impact of biopesticides. Australia's regulatory process is known for its thoroughness and transparency, but this also means longer approval times and higher costs for developers [197].

The regulatory challenges for PGPR commercialization vary significantly across different regions. While stringent regulations ensure safety and efficacy, they also pose barriers to market entry. Harmonizing regulations and streamlining approval processes can help overcome these challenges, promoting the global adoption of PGPR for sustainable agriculture.

### 3.2. Successful Case Studies of PGPR Commercialization

In the Netherlands, *Pseudomonas fluorescens* has been successfully commercialized for use in wheat cultivation. This PGPR strain enhances plant growth by producing the antibiotic 2,4-diacetyl phloroglucinol, which suppresses various soilborne pathogens. Its application has led to healthier plants and higher wheat yields [10, 198]. The Chinese Academy of Agricultural Sciences has made significant strides in developing and commercializing PGPR-based biostimulants. These products have been used to enhance nutrient uptake, improve plant disease resistance, and increase crop yields in various agricultural systems. The biostimulants are particularly effective in organic farming and sustainable agriculture practices [199]. The isolation and commercialization of *Bacillus* spp. in the Andaman Islands for managing bacterial wilt in eggplants have been successful. The use of *Bacillus* strains has significantly reduced disease prevalence and improved overall crop health and productivity [200]. These case studies underscore the importance of tailored strategies for the successful commercialization of PGPR. Key factors include the selection of effective strains, rigorous field trials, formulation development, and farmer education to ensure adoption.

## 4. Isolation and Identification of PGPR

Before conducting *in vitro* characterization assays of PGPR in the laboratory and subsequent field trials, PGPR strains must first be isolated and identified from their natural habitats. The bulk soil and rhizosphere contain many microbes other than the desired PGPR. Therefore, the isolation method must be designed to favor the desired PGPR over other rhizosphere microbes. In addition, these identification methods must be rapid to quickly test as many microorganisms as possible. Identification of PGPR based solely on biochemical and physiological features has sometimes proven inconclusive; therefore, microbial identification is only definitive using advanced molecular tools such as 16S rDNA gene sequencing [201]. The RDP (Ribosomal Database Project) and NCBI (National Center for Biotechnology Information) provide comprehensive databases for sequenced microbial strains, offering a wealth of information for PGPR identification. In addition to the commonly used 16S rDNA sequencing, other species-specific molecular tools such as RAPD (random amplified polymorphic DNA), MLSA (multilocus sequence analysis), and restriction length polymorphism are used in PGPR identification [202].

### 4.1. Selection and Characterization of PGPR

Characterization, a series of *in vitro* assays conducted in the laboratory, is used to predict the biocontrol potential of rhizosphere bacterial strains. However, the selection process, which is based on strain performance in the field's highly unpredictable abiotic environmental conditions, presents a real challenge. This process is laborious and cannot be performed on many strains simultaneously. Despite promising *in vitro* characterization assay results, the field performance of such PGPR is not guaranteed. To address this, a solution has been proposed: the selection process should commence with greenhouse screening [203]. Many scientific reports have described PGPR strains as plant- and soil-specific, which limits their broad-spectrum efficacy.

### 4.2. Consortium and Synthetic Community of PGPR

While several PGPR strains that showed promise during *in vitro* testing later performed poorly in field trials due to harsh abiotic conditions, their potential can be harnessed in a consortium. This agricultural application could potentially alleviate the problem, although the effect on efficacy can be neutral, positive, or negative. A typical example of a positive synergistic consortia effect is a combination treatment that helped regress tomato wilt disease development [204]. Similarly, it was demonstrated that the synergistic use of *Pseudomonas fluorescen*s and *Bacillus subtilis* improves plant growth [205]. In-depth knowledge of plant-microbe interactions and PGPR modes of action is required to efficiently adopt biological control in disease management. There are still gaps in our knowledge, and these drawbacks have limited the development and marketing of PGPR as biocontrol agents.

Another emerging technique is the synthetic community (SynCom) approach. SynCom involves incorporating synthetic biology approaches to generate a microbial consortium. Several studies have reported that SynCom application significantly enhanced the growth of various plants, such as maize and sorghum, underscoring its potential [206, 207]. While the SynCom approach is promising, it is not without challenges. Designing SynComs with hundreds of microbes is daunting, given the difficulties of handling them.

## 5. Future Research Direction

Despite significant advances in PGPR research over the last three decades, numerous gaps in knowledge remain. Addressing these gaps is critical to enhancing the commercial viability and efficacy of PGPR bioproducts. There is an urgent need for improved communication between public researchers and the industry to facilitate the commercialization of PGPR bioproducts. Enhancing collaboration can bridge the gap between laboratory research and field application, ensuring that bioproducts are both effective and economically viable for growers. Significant knowledge gaps exist in the production, formulation, and delivery of PGPR bioproducts. Research should focus on optimizing these processes to increase the commercial success of PGPR bioproducts. Developing robust and consistent methods for producing and formulating PGPR can improve product stability, shelf life, and field efficacy.

Understanding how PGPR ameliorates abiotic stress in plants, including the identification of involved biomolecules and biosynthesis pathways, is crucial. Research in this area can lead to the development of PGPR strains that enhance plant resilience to environmental stresses, thereby improving crop yields and sustainability. Future research should also focus on the molecular interactions between microbes in consortia and the interactions between host plants and soil microbes. This understanding can lead to the development of more effective microbial consortia that enhance plant growth and disease resistance.

There is a need to improve the methods used for quantifying the efficacy and presence of PGPR in bioproducts. Developing accurate and reliable quantification techniques can help in assessing the performance of PGPR bioproducts in the field and ensuring consistent application rates. The enzymes and pathways involved in the bacterial biosynthesis of phytohormones are still not fully understood. Researching these pathways can provide insights into how PGPR promotes plant growth and can lead to the development of strains with enhanced growth-promoting capabilities.

Finally, more research is required to successfully integrate PGPR-based products into existing crop systems. This includes developing strategies for alternating between chemical and PGPR products and creating forecast models to determine the optimal timing for applying these products. Such integration can improve the sustainability and effectiveness of agricultural practices.

## 6. Conclusion

The exploration of plant growth-promoting rhizobacteria (PGPR) reveals their profound potential in advancing sustainable agriculture through various direct and indirect mechanisms. These beneficial bacteria enhance plant growth, increase nutrient availability, produce vital phytohormones, and provide robust protection against phytopathogens. Moreover, PGPR plays a pivotal role in improving plant tolerance to abiotic stresses such as salinity, drought, and heavy metal contamination, thereby contributing to overall plant health and productivity. The integration of PGPR into agricultural practices holds the promise of reducing the reliance on synthetic chemicals, thus mitigating environmental impact and promoting sustainable farming practices. By leveraging the natural capabilities of PGPR, we can pave the way for a greener, more resilient agricultural future. This endeavor necessitates a concerted effort from researchers, industry stakeholders, and policymakers to harness the full potential of PGPR for the benefit of global agriculture and environmental sustainability.

However, the path to widespread commercialization and adoption of PGPR-based products is fraught with challenges. These include the specificity of PGPR strains to particular crops, the formulation stability, and the acceptance by farmers accustomed to the immediate results of chemical fertilizers and pesticides. Addressing these challenges requires continuous research and development, improved formulation techniques, and robust field trials to demonstrate the efficacy and reliability of PGPR products under diverse agricultural conditions. The next steps for researchers involve focusing on identifying and optimizing PGPR strains for multiple crops and conditions, elucidating detailed mechanisms of action, conducting large-scale field trials, and integrating biotechnology advancements. Industry practitioners should concentrate on developing stable, cost-effective PGPR products, providing farmer training and support, ensuring regulatory compliance, and promoting PGPR as part of integrated pest management and sustainable farming practices. By addressing these areas, researchers and industry practitioners can fully harness the potential of PGPR to advance sustainable agriculture, reduce reliance on synthetic chemicals, and promote environmental sustainability.

## Figures and Tables

**Table 1:** Mode of actions by which PGPR stimulate plant growth.

PGPR forms	Definition	Mode of action	References
Phytostimulator	These are a group of rhizobacteria with a unique ability to produce phytohormones, e.g., cytokinin, indole acetic acid (IAA), and gibberellin	Production of phytohormones	[20–22]

Biofertilizer	These are living organisms or products made from living organisms, especially microbes when applied to a plant colonize the root microenvironment, thereby stimulating increased growth of the plant through increased bioavailability of essential nutrients to the host plant	Solubilization of inorganic phosphates	[23]
		Nitrogen fixation	[24–26]

Biopesticide	These are microbes that help promote plant growth by antagonism of phytopathogens that are harmful to the plant	Induced and acquired systemic resistance	[27–30]
		Lytic enzyme production	[31–34]
		Production of siderophore, antibiotic, hydrogen cyanide (HCN)	[35–37]

**Table 2:** Biocontrol of selected PGPR strains against known phytopathogens and the host plants from which they were isolated.

PGPR strains	Diseases/pathogens/benefits	Host plant(s)	References
*Actinobacteria*	*Penicillium chrysogenum, Aspergillus niger,* and *Microsporum gypseum*	Common wheat (*Triticum aestivum*)	[99]
*Achromobacter piechaudii* ARV8	Salt stress resistance	Tomato (*Solanum lycopersicum* L)	[100]
*Azospirillum brasilense*	Virulent rhizosphere fungi	Cherry plum (*Prunus cerasifera* L.)	[101]
*Azotobacter chroococcum*	Solubilization of inorganic phosphate	Wheat *(Triticum aestivum*)	[102]
*Bacillus amyloliquefaciens*	Tomato mottle virus transmitted by (*Myzus persicae* Sulzer)	Tomato (*Solanum lycopersicum* L) and bell pepper (*Capsicum annuum*)	[103]
*Bacillus circulans*	Inorganic phosphate solubilisation	Mung bean (*Vigna radiata*)	[104]
*Bacillus cereus and Bacillus subtilis*	*Rhizoctonia solani* AG 8	Common wheat (*Triticum aestivum*)	[105]
*Bacillus megaterium*	Induction of systemic resistance	Tea (*Camellia sinensis*)	[74]
*Bacillus subtilis* G803	*Myzus persicae*	Pepper (*Piper nigrum*)	[106]
*Bacillus subtilis* CE1	*Fusarium verticilloides*	Maize (*Zea mays*)	[107]
*Bacillus amyloliquefaciens B9601-Y2*	Leaf mildew	Tobacco (*Nicotiana tabacum*)	[108]
*Bacillus polymyxa*	Inorganic phosphate solubilisation	Mung bean (*Vigna radiata*)	[109]
*Bacillus firmus*	Inorganic phosphate solubilisation	Wheat *(Triticum aestivum*)	[110]
*Bacillus licheniformis*	*Myzus persicae*	Pepper (*Piper nigrum*)	[111]
*Bradyrhizobium japonicum*	Inorganic phosphate solubilisation	Soybeans (*Glycine max*)	[112]
*Brevibacillus* sp.	*Corynespora cassiicola*	Sleeping plant (*Phyllanthus amarus*)	[113]
*Brevundimonas olei* Prd2	Fusarium wilt	Tomato (*Solanum lycopersicum* L)	[113]
*Klebsiella sp.*	Amelioration of water stress	Wetland rice (*Oryza sativa*)	[114]
*Pseudomonas aeruginosa*	Root rot	Mung bean (*Vigna radiata*)	[115]
*Pseudomonas fluorescens* MKB 249	*Fusarium culmorum*	Wheat (*Triticum aestivum*) and barley (*Hordeum vulgare*)	[116]
*Pseudomonas syringae*	Tobacco necrosis virus	Tobacco (*Nicotiana tabacum)*	[117]
*Pseudomonas chlororaphis*	*Macrophomina phaseolina*	Sorghum (*Sorghum bicolor*)	[118]
*Pseudomonas chlororaphis*	Inorganic phosphate solubilisation	Soybeans (*Glycine max*)	[119]
*Pseudomonas fluorescence* LRB3W1	*Fusarium oxysporum f. sp. lycopersici*	Tomato (*Solanum lycopersicum* L)	[120]
*Pseudomonas* sp RhB-12	*Fusarium oxysporum f. sp. lycopersici*	Tomato (*Solanum lycopersicum* L)	[121]
*Rhizobium leguminosarum*	Biological nitrogen fixation	Beans (*Phaseolus vulgaris*)	[122]
*Serratia marcescens*	Amelioration of salt stress	Common wheat (*Triticum aestivum)*	[123]

**Table 3:** A detailed outline of some selected commercially available PGPR strains in the market.

PGPR strains	Pathogen/disease/benefits	Target plant(s) benefits	EPA/EU approval no	Product/company/country	References
*Agrobacterium radiobacter strain* K84	*Agrobacterium tumefaciens*	Oranges	11,4201	AgBioChem, USA	[163]
*Pseudomonas fluorescens* A506	Blight and rot	Potato, tomato, and fruits	00,6438	Plant Health Technologies, USA	[164]
*Streptomyces griseoviridis strain* K61	Soilborne pathogen	Seedlings of trees, ornamental plants, and fruits	12,9069	Kemira Oy, Finland	[165]
*Bacillus subtilis* MBI 600	Alternaria, fusarium, aspergillus, and rhizoctonia	Soybeans, cotton, maize, peanuts, beans, peas, wheat, and barley	12,9082	Becker Underwood; Premier Horticulture, USA	[166]
*Pseudomonas syringae strain* ESC-10	Storage diseases	Oranges, pears, grapefruit, or lemon	00,6441	EcoScience Produce Systems Division, USA	[167]
*Agrobacterium radiobacter strain* K1026	*Agrobacterium rhizogenes*	Fruits	00,6474	Bio-Care Technology, Australia	[168]
*Pseudomonas aureofaciens strain* Tx-1	*Pythium aphanidermatum*	Recreational turf	00,6473	Eco Soil Systems, USA	[169]
*Bacillus subtilis* var. *amyloliquefaciens strain* FZB24	Fusarium and rhizoctonia	Tomatoes, shrubs, and trees	00,6480	Earth Biosciences, USA	[170]
*Bacillus subtilis strain* QST 713	Blight and rot	Peppers, tomatoes, cucurbits, leafy vegetables, grapes, walnut, potatoes, and cherries	00,6479	AgraQuest, USA	[171]
*Pseudomonas chlororaphis strain* 63-28	*Rhizoctonia solani* and Pythium	Pepper	00,6478	Eco Soil Systems, USA	[172]
*Azotobacter chroococcum*	Nitrogen fixation	Legumes	N/A	Manidharma, India	[173]
*Pseudomonas chlororaphis*	*Fusarium* sp.	Cereals	540/2011	Cedomon™, Sweden	[174]
*Bacillus subtilis strain QST713*	Early blight	Potato, pepper, and grapes	006479	Rhapsody, USA	[175]
*Bacillus cereus strain UW85*	Pythium	Cucumber	007173	Pix Plus Plant Regulator, USA	[13]
*Bacillus amyloliquefaciens strain D747*	Broad spectrum against greenhouse diseases	Cucumber, tomato, tobacco, etc.	70051-10	Amylo-X®, UK	[176]

## Data Availability

The data from the literature that support the findings of this study are fully presented within the article.
